# Dynamic Regulation of GH–IGF1 Signaling in Injury and Recovery in Hyperoxia-Induced Neonatal Lung Injury

**DOI:** 10.3390/cells10112947

**Published:** 2021-10-29

**Authors:** Christina Vohlen, Jasmine Mohr, Alexey Fomenko, Celien Kuiper-Makris, Tiffany Grzembke, Rabia Aydogmus, Rebecca Wilke, Dharmesh Hirani, Jörg Dötsch, Miguel A. Alejandre Alcazar

**Affiliations:** 1 Department of Pediatric and Adolescent Medicine, Translational Experimental Pediatrics—Experimental Pulmonology, Faculty of Medicine and University Hospital Cologne, University of Cologne, 50937 Cologne, Germany; Christina.Vohlen@uk-koeln.de (C.V.); Jasmine.mohr@uk-koeln.de (J.M.); Alexey.Fomenko@uni-duesseldorf.de (A.F.); centina.kuiper-makris@uk-koeln.de (C.K.-M.); Tiffany.Grzembke@gmail.com (T.G.); raydogmu@smail.uni-koeln.de (R.A.); rebecca.wilke@uk-koeln.de (R.W.); dharmesh.hirani@uk-koeln.de (D.H.); 2 Department of Pediatric and Adolescent Medicine, Faculty of Medicine and University Hospital Cologne, University of Cologne, 50937 Cologne, Germany; joerg.doetsch@uk-koeln.de; 3 The German Centre for Lung Research (DZL), Institute for Lung Health, University of Giessen and Marburg Lung Centre (UGMLC), Justus-Liebig University Gießen, 35392 Gießen, Germany; 4 Center for Molecular Medicine Cologne (CMMC), Faculty of Medicine and University Hospital Cologne, University of Cologne, 50937 Cologne, Germany; 5 Cologne Excellence Cluster for Stress Responses in Ageing-Associated Diseases (CECAD), Faculty of Medicine and University Hospital Cologne, University of Cologne, 50937 Cologne, Germany

**Keywords:** neonatal hyperoxia and bronchopulmonary dysplasia, growth hormone and IGF1, lung injury and regeneration, alveolar epithelial type II cell, neonatal lung fibroblast

## Abstract

Prematurely born infants often require supplemental oxygen that impairs lung growth and results in arrest of alveolarization and bronchopulmonary dysplasia (BPD). The growth hormone (GH)- and insulin-like growth factor (IGF)1 systems regulate cell homeostasis and organ development. Since IGF1 is decreased in preterm infants, we investigated the GH- and IGF1 signaling (1) in newborn mice with acute and prolonged exposure to hyperoxia as well as after recovery in room air; and (2) in cultured murine lung epithelial cells (MLE-12) and primary neonatal lung fibroblasts (pLFs) after treatment with GH, IGF1, and IGF1-receptor (IGF1-R) inhibitor or silencing of GH-receptor (*Ghr*) and *Igf1r* using the siRNA technique. We found that (1) early postnatal hyperoxia caused an arrest of alveolarization that persisted until adulthood. Both short-term and prolonged hyperoxia reduced GH-receptor expression and STAT5 signaling, whereas *Igf1* mRNA and pAKT signaling were increased. These findings were related to a loss of epithelial cell markers (SFTPC, AQP5) and proliferation of myofibroblasts (αSMA^+^ cells). After recovery, GH-R-expression and STAT5 signaling were activated, *Igf1r* mRNA reduced, and SFTPC protein significantly increased. Cell culture studies showed that IGF1 induced expression of mesenchymal (e.g., *Col1a1*, *Col4a4*) and alveolar epithelial cell type I (*Hopx*, *Igfbp2*) markers, whereas inhibition of IGF1 increased SFTPC and reduced AQP5 in MLE-12. GH increased *Il6* mRNA and reduced proliferation of pLFs, whereas IGF1 exhibited the opposite effect. In summary, our data demonstrate an opposite regulation of GH- and IGF1- signaling during short-term/prolonged hyperoxia-induced lung injury and recovery, affecting alveolar epithelial cell differentiation, inflammatory activation of fibroblasts, and a possible uncoupling of the GH-IGF1 axis in lungs after hyperoxia.

## 1. Introduction

Prematurely born infants often require mechanical ventilation (MV) and/or supplemental O_2_, which are life-saving treatments that, however, elicit a chronic form of lung injury called bronchopulmonary dysplasia (BPD). BPD is characterized by reduced formation of alveoli and perturbed extracellular matrix remodeling [1,2,3]. While current treatment options are mainly supportive, molecular strategies and effective curative therapies to reverse lung pathology as well as impaired lung function remain elusive. Therefore, understanding the molecular regulation of alveolar formation and regeneration after injury is of high clinical importance in order to develop effective strategies for patients’ suffering from BPD.

The growth hormone (GH) and insulin-like growth factor 1 (IGF1) axis is a principle endocrine system predominantly regulating body growth during childhood and anabolism [4,5]. IGF1 is the primary effector of GH that binds to the IGF1 receptor (IGF1-R), activating the receptor tyrosine kinase and thereby initiating AKT signaling [6]. Interestingly, a recent study showed that postnatal deletion of *Igf1r* caused alveolar simplification and perturbed lung matrix remodeling [7]. Similarly, transgenic IGF1 deficient mice are characterized by a decreased viability due to alveolar hypoplasia, causing respiratory distress [8]. From a cell-specific perspective, there is a growing body of evidence suggesting that IGF1 plays a role in the regulation of proliferation and differentiation of lung epithelial cells as well as fibroblasts, thereby contributing to lung repair processes [6]. Alveolar epithelial type II cells (ATIIs) are lung progenitor cells, accountable for the significant and physiological regeneration capacity of the lung. ATIIs have the ability to self-renew and give rise to ATIs after lung injury. Furthermore, the repair of damaged alveoli is mediated via ATII–fibroblast interactions, which are tightly coordinated in lung development [9].

Mediators of the IGF1 signaling axis have been postulated as biomarkers for lung diseases, in various studies, since they are locally and/or systemically dysregulated [6]. For example, acute and chronic lung diseases are associated with an upregulation of IGF1 and IGF1-R [10,11,12]. On the other hand, in preterm infants, studies have linked a decrease in IGF1 to BPD, whereas treatment with rhIGF1/BP3 has been shown to improve lung growth in experimental BPD [13]. Previous studies of our group showed a dynamic regulation of the GH–IGF1 signaling cascade in lungs after intrauterine growth restriction. While an intrauterine inhibition of GH–IGF1 was associated with disturbed pulmonary growth, postnatal activation was linked to catch-up growth of the lung [4]. Based on prior studies, we investigated if GH–IGF1 signaling is disrupted in lungs of newborn mice exposed to short and prolonged hyperoxia as well as after recovery in normoxia. In addition, we studied the impact of GH–IGF1 on cultured murine lung epithelial cells (MLE-12) and primary lung fibroblasts. Here, we show that lung intrinsic GH–IGF1 signaling is dynamically regulated during lung injury and recovery after hyperoxia. Our data demonstrate that neonatal hyperoxia results in an upregulation of *Igf1* gene expression and an activation of AKT signaling, whereas GH–STAT5 signaling was reduced during the acute injury phase. After recovery in normoxia, however, GH receptor (GH-R) protein was increased, and STAT5 signaling was activated. Moreover, in vitro experiments showed that stimulation of MLE-12, as a model of ATII cells, with IGF1 increased the expression of mesenchymal and ATI markers, while GH had no effect. In primary lung fibroblasts, GH induced the expression of IL6 and proliferation, whereas IGF1 had the opposite effect. Therefore, our data support the notion that GH–IGF1 signaling could represent a potential target to develop novel therapeutic strategies to treat preterm infants with BPD.

## 2. Materials and Methods

### 2.1. Animal Studies

#### 2.1.1. Experimental Setting for Hyperoxia-Induced Lung Injury

All animal procedures were performed in accordance with German regulations and legal requirements and were approved by the local government authorities (LANUV, NRW, Düsseldorf, Germany; AZ. AZ.2015.A120). Adult and neonatal male and female C57BL/6N mice were housed in humidity- and temperature-controlled chambers, exposed to a 12 h dark/light cycle, and were allowed food and water ad libitum. Newborn pups from 4 to 7 litters were randomly pooled and equally distributed between dams on the day of birth. Litters were either exposed to 85% (vol/vol) O_2_ (HYX) or maintained at room air (21% (vol/vol) O_2_; normoxia, NOX) as previously described [14]. Nursing dams were rotated between HYX and NOX litters every 24 h to exclude toxic O_2_-related effects on the dams. Control litters were maintained in room air throughout life, while the experimental group was exposed to HYX from postnatal day 1 (P1) to P28. Exposure to 85% O_2_ was performed in a 90 × 42 × 38 cm Plexiglas chamber. O_2_ concentrations were monitored using a miniox II monitor (Catalyst Research, Owing Mills, MD, USA). Animals were deeply anesthetized via intraperitoneal injection of xylazine (10 mg/kg body weight) and ketamine (100 mg/kg body weight) in sodium chloride (NaCl 0.9%) solution and sacrificed by cardiac puncture at P7, P28, and P70.

#### 2.1.2. Physiological Data of the Offspring

Body weight and lung weight (g) of pups were measured at different time points: P7, P28, and P70 (Table 1).

#### 2.1.3. Tissue Preparation and Postmortem Processing of the Lungs

The lungs were excised and snap-frozen in liquid nitrogen or pressure-fixed at P28 and P70 for mRNA and protein analysis or histomorphometric analyses, respectively. To this end, the right lobe of lungs was removed after ligation of the bronchus and immediately snap-frozen in liquid nitrogen an dstored at −80 °C for further molecular assays. Subsequently, the left lobe was inflated via tracheotomy and pressure-fixed at 20 cm H_2_O with 4% (mass/vol) paraformaldehyde (PFA), and the trachea was ligated. Lungs were excised, submersed in 4% (mass/vol) PFA overnight, followed by paraffin embedding for sectioning.

#### 2.1.4. Quantitative Histomorphometric Analysis of Lung Sections

Paraffin sections (3 μm) were mounted on poly-l-lysine-coated glass slides, dewaxed with xylene (3–5 min) and rehydrated in a graduated series of ethanol solutions [100%, 95%, and 70% (vol/vol)] and then water. For quantitative histomorphometric analysis, lung sections were stained with hematoxylin & eosin [14,15], and analyzed using light microscopy (20× magnification), a slide scanner (Leica SCN400, Wetzlar, Germany), and Cell D 3.4 Olympus soft image solutions (Olympus, Hamburg, Germany). We assessed three parameters indicating alveolar size and number as previously described [14,15]: radial alveolar count (RAC), average alveolar surface of a single alveolus, and mean linear intercept (MLI). We analyzed ten random fields of view per section and a total of 3–4 animals per group.

### 2.2. Cell Culture Experiments

#### 2.2.1. Murine Alveolar Epithelial Cells Type II (MLE-12)

Transformed mouse lung epithelial cells (MLE-12) (#CRL-2110, ATCC, Manassas, VA, USA) were cultured in DMEM/F-12 (#11039021, Thermo Fisher Scientific, Waltham, MA, USA) medium supplemented with 10 nM mL^−1^ β-estradiol, 10 nM mL^–1^ hydrocortisone, 2% FBS, 1% penicillin–streptomycin solution, and 1% insulin–transferrin–selenium (ITS-X) (serum-rich medium). For starvation, MLE-12 cells were cultured in DMEM/F-12 medium supplemented with 10 nM mL^−1^ β-estradiol, 10 nM mL^–1^ hydrocortisone, 0.2% FBS, 1% penicillin–streptomycin solution, and 1% insulin–transferrin–selenium (ITS-X) (serum-reduced medium). Cells were cultured in a humidified incubator under normal growth conditions of 5% CO_2_ at 37 °C. MLE-12 cells are a model of ATII cells expressing the specific markers *SftpB* and *SftpC*. Cells were split 1:10 twice a week and used for experiments between passages 3 and 12. Three to six independent experiments were performed for each assay as well as for RNA and protein studies.

#### 2.2.2. Murine Primary Lung Fibroblasts (pLFs)

Isolation of pLFs from C57BL/6N mice was performed on P12 as described previously [4]. Then, pLFs were cultured in DMEM medium (#41966-029, Thermo Fisher Scientific, Waltham, MA, USA), supplemented with 1% penicillin–streptomycin solution and with 10% FBS. For starvation, pLFs were cultured in DMEM medium supplemented with 1% penicillin–streptomycin solution, and 1% FBS (serum-reduced medium).

#### 2.2.3. Rat Primary Neonatal Lung Myofibroblasts (pnFs)

Primary neonatal lung myofibroblasts (pnFs) were isolated at P3 from rats using a one-step incubation procedure as previously described [4,16] and subsequently cultured in DMEM medium (Gibco; Thermo Fischer Scientific; #41966-029; Waltham, MA, USA) with 10% FBS. All experiments were performed with cells in passages 2 to 6.

#### 2.2.4. Exposure of MLE-12 and pLFs to HYX

MLE-12 and murine primary lung fibroblasts (pLFs) were seeded and kept in normoxic conditions (21% O_2_; NOX), while the experimental groups were exposed to 85% O_2_ (HYX) for 24 h (gene expression analysis) or 48 h (protein expression analysis) in serum-rich medium (2% FBS for MLE-12, 10% for pLF). For RNA and protein extraction, cells were harvested in Dulbecco’s phosphate-buffered saline (DPBS, #14190094, Thermo Fisher Scientific, Waltham, MA, USA) using a cell scraper.

#### 2.2.5. Treatment with GH, IGF1, and IGF1-R Inhibitor

GH (#86900, Sigma-Aldrich, St. Louis, MO, USA), IGF1 (#I8779 Sigma-Aldrich, St. Louis, MO, USA), and picropodophyllin (IGF1-R inhibitor; #S7668 Selleckchem, München, Germany) were used to analyze the role of GH–IGF1 signaling in MLE-12 and pLFs. First, 0.5–1.0 × 10^6^ cells were seeded per 10 cm dish in serum-rich medium (2% FBS for MLE-12, 10% for pLF) and incubated under normal growth conditions for 24 h (5% CO_2_ at 37 °C). Prior to stimulation with GH and IGF1, cells were starved for 12 h using serum-reduced medium (0.2% FBS for MLE-12, 1% for pLF). Subsequently, cells were stimulated with GH (100 nmol/L or 200 nmol/L) diluted in 10 mmol sodium bicarbonate, IGF1 (10 ng/mL or 100 ng/mL) diluted in dH_2_O, and IGF1-R inhibitor (20 nM/mL) diluted in ethanol. Controls were treated with the respective vehicle. Six independent experiments were performed; for mRNA or protein isolation, cells were collected 24 h after stimulation.

#### 2.2.6. siRNA Transfection Experiments

Cultured rat pnF cells were transfected with either anti *Ghr*-siRNA (Dharmacon; OnTarget Smartpool Plus; #L-090388-02-005; Lafayette, CO, USA), anti *Igf1r*-siRNA (Dharmacon; OnTarget Smartpool Plus; #L-091936-02-0005; Lafayette, CO, USA), or scrambled siRNA (scr) (ON-TARGETplus Non-targeting Control Pool; #D-001810-10-05; Lafayette, CO, USA) using Lipofectamine^TM^ 2000 (Invitrogen; #11668027; Waltham, MA, USA). Briefly, pnF were grown to 60–70% confluency in serum-rich medium, followed by exposure to either siRNA against *Ghr* (si-*Ghr*), *Igf1r* (si-*Igf1r*), or control (scrambled (scr) siRNA) in Opti-MEM (Life Technologies, cat no. 31985-070) for 4 h. Cells were maintained in serum-rich medium and harvested after 72 h to ensure sufficient time to suppress GH-R and IGF1-R expression. Protein was isolated for immunoblots.

#### 2.2.7. Viability Assay

An MTT assay (ATCC, cat. no. 30-1010K) was used to assess cell viability. Cells were seeded in a 96-well plate (5000 cells per well) in serum-rich medium, followed by stimulation with GH or IGF1 stimulation for 24 h. In order to assess cell viability after silencing *Ghr* and *Igf1r*, cells were transfected with si-*Ghr*, si-*Igf1r*, or scr siRNA for 48–72 h, followed by exposure to serum-rich medium or serum-reduced medium overnight and subsequent incubation for 2 h with 10 μL of yellow tetrazolium MTT (3-[4,5-dimethylthiazolyl-2]-2,5-diphenyltetrazolium bromide), which is reduced by metabolically active cells. Absorbance (570 nM) was measured in a plate reader (Tecan).

#### 2.2.8. Proliferation Assays (Bromodeoxyuridine (BrdU) Incorporation Assay)

MLE-12 and pLFs were seeded into 96-well plates and grown for 24 h in serum-rich medium (2% FBS for MLE-12, 10% for pLF). Then, cells were stimulated with GH (100 nmol/L or 200 nmol/L) diluted in 10 mmol sodium bicarbonate or IGF1 (10 ng/mL or 100 ng/mL) diluted in dH_2_O. Controls were treated with the respective vehicle. Bromodeoxyuridine (BrdU) labeling solution was added when the cells were 80% confluent and subsequently cultured in serum-rich medium (2% FBS for MLE-12, 10% for pLF) for 24 h. At the end of the experiment, incorporation of BrdU was assayed using a BrdU cell proliferation assay kit (#11647229001, Roche, Basel, Switzerland) according to the manufacturer’s instructions.

### 2.3. Enzyme-Linked Immunosorbent Assay (ELISA)

Measurement of IL-6 concentrations in the cell culture supernatants of MLE-12 and pLFs after exposure to GH, IGF1, or the respective vehicle for 24 h was performed according to the protocol of commercially available ELISA systems (mouse IL6 uncoated ELISA (Invitrogen; Waltham, MA, USA) #88-7064).

#### 2.3.1. Immunoblotting

Immunoblotting was performed with protein isolated from whole lung homogenates, MLE-12, pnFs or pLFs as previously described [17]. Blots were incubated with the following antibodies: anti-rabbit pSTAT5 (#4322, 1:1000, Cell Signaling, Danvers, MA, USA), anti-rabbit STAT5 (#94205, 1:2000, Cell Signaling, Danvers, MA, USA), anti-rabbit pAKT (#4058, 1:1000, Cell Signaling, Danvers, MA, USA), anti-rabbit AKT (#9272, 1:2000, Cell Signaling, Danvers, MA, USA), anti-rabbit growth hormone receptor (GH-R) (#NBP1-95983, 1:1000, Novus Biologicals, Littleton, CO, USA), anti-rabbit insulin-like growth factor receptor 1 (IGF1-R) (#3027, 1:1000, Cell Signaling, Danvers, MA, USA), anti-mouse proliferating cell nuclear antigen (PCNA) (#M0879, Aligent Dako, Santa Clara, CA, USA), anti-mouse aquaporin 5 (AQP5) (#A4979, 1:2000, Sigma-Aldrich, St. Louis, MO, USA), and anti-mouse surfactant protein C (SFTPC) (#AP3786, 1:2000, Millipore, Burlington, MA, USA), with anti-mouse β-actin (#3700, 1:5000, Cell Signaling, Danvers, MA, USA) serving as the loading control. Anti-mouse IgG, HRP-linked (#7076, Cell Signaling, Danvers, MA, USA) and anti-rabbit IgG, HRP-linked (#7074, Cell Signaling, Danvers, MA, USA) were used as secondary antibodies.

#### 2.3.2. Immunofluorescent Staining

In order to assess proliferative myofibroblasts in lungs after hyperoxia, immunofluorescent staining for Ki67 (proliferative marker) and αSMA (marker of myofibroblasts) was performed as described previously [18]. Briefly, lung sections were deparaffinized, rehydrated, and then treated with MaxBlock™ reagent A (MaxVision Biosciences, #MB-L, Washington, DC, USA) for 5 min at RT; the slides were washed with 60% ethanol for 1 min and 5 min with PBS. For antigen retrieval, lung sections were exposed to 10 mM citrate buffer (pH 6; Dako, #S2369, Germany) at 90–120 °C for 25 min. Subsequently, tissue sections were treated with blocking solution (Sea Block, Thermo Scientific™, #37527, The Netherlands) at RT for 1 h. Lung sections were incubated with the primary antibody mouse anti Ki67 (Invitrogen, #PA5-19462, The Netherlands) and mouse anti-αSMA-CY3 (Sigma-Aldrich, #C6198, USA, 1:200) in the dark at 4 °C overnight. DAPI dye Sigma-Aldrich, #D9542, Germany, 1:5000) was used to counterstain the nucleus. Lung sections were then treated with Post-Detection Conditioner Reagent B (MaxVision Biosciences, #MB-L, Washington, DC, USA) for 5 min at RT, and then slides were mounted in fluoromount aqueous (Sigma Aldrich, #F4680, St. Louis, MO, USA). Images of lung sections were taken using a fluorescence microscope (Olympus, Hamburg, Germany) at 40× and 100× magnification. Four fields of view per lung section and four animals per group were analyzed. We quantified αSMA^+^ cells relative to all DAPI^+^ cells (%) as well as relative number of Ki67^+^ and αSMA^+^ cells (%).

#### 2.3.3. RNA Extraction, Real-Time RT-PCR

Total RNA was isolated from whole lung homogenate, MLE-12, or pLFs using TriReagent, and real-time RT-PCR was performed as described previously, using the 7500 real-time PCR system (Applied Biosystem, Foster City, CA, USA) [17]. The relative amount of the specific mRNA was normalized to *Actb* (β-actin). Primers and Taq-Man probes were designed using Primer Express Software and are listed in Supplementary Table S1.

### 2.4. Statistics

For statistical analyses, an unpaired *t*-test, paired *t*-test, and RM one-way ANOVA with Bonferroni post-test were performed. Significances were indicated with * *p* < 0.05, ** *p* < 0.01, and *** *p* < 0.001 in the graphs representing the mean value ± standard error of the mean (SEM). Data were graphically represented using GraphPad Prism 7 software (GraphPad Software, LLC, San Diego, CA, USA).

## 3. Results

### 3.1. HYX Induces an Arrest of Alveolarization That Persists in Adulthood

**Figure 1:**
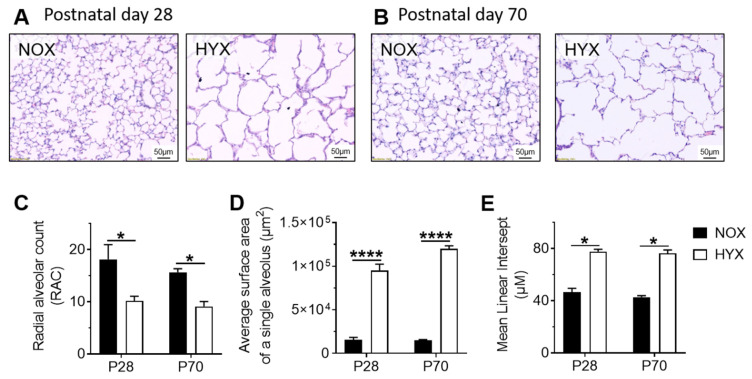
Quantitative histomorphometric analysis of lungs of mice at postnatal day 28 (P28) and P70. Mice were exposed to hyperoxia (HYX, 85% O_2_) from birth until P28, followed by recovery in room air (normoxia, NOX, 21% O_2_); control mice were always in room air. (**A**,**B**) Representative hematoxylin & eosin stained lungs at P28 (**A**) and P70 (**B**). (**C**–**E**) Quantitative histomorphometry at P28 and P70 using three parameters of alveolar growth: radial alveolar count (RAC) (**C**), average surface of a single alveolus (**D**), and mean linear intercept (MLI) (**E**). Data are presented as mean ± SEM; * *p* < 0.05, **** *p* < 0.0001 (unpaired *t* test); *n* = 3–4/group.

First, quantitative histomorphometric analyses of lungs of mice exposed to prolonged hyperoxia (HYX) from birth until P28 confirmed a marked arrest of alveolarization. We found a significant reduction in RAC along with an increase of the average surface area of a single alveolus and of the MLI in lungs after HYX when compared to lungs of mice exposed to room air (normoxia, NOX), indicating larger and fewer alveoli. We next investigated if these structural changes persist in adulthood after recovery in room air. The mice were transferred from HYX to NOX at P28 and remained in room air until P70. Assessment of lung structure at P70 showed that RAC remained reduced, and both average alveolar surface of a single alveolus and MLI increased. These findings demonstrate that HYX-induced neonatal lung injury persists in adulthood, suggesting impaired repair mechanisms and reduced regenerative capacity of the lung (Figure 1A–E).

### 3.2. HYX Dynamically Regulates the GH–IGF1 Axis during Acute and Chronic Lung Injury

**Figure 2:**
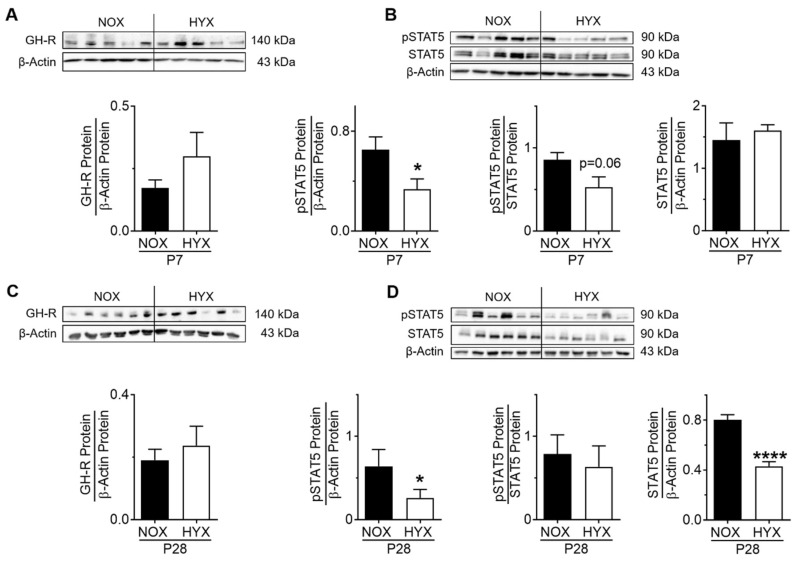
Growth hormone (GH) signaling in lungs of mice exposed to room air (normoxia, NOX, 21% O_2_) or hyperoxia (HYX, 85% O_2_) from birth until postnatal day 7 (P7) or P28. (**A**,**C**) Assessment of GH receptor (GH-R) protein abundance in lungs of mice exposed to NOX or HYX at P7 (**A**) and P28 (**C**). (**B**,**D**) Analysis of phosphorylation of signal transducer and activator of transcription 5 (STAT5) protein in lungs of mice exposed to NOX or HYX at P7 (**B**) and P28 (**D**). Densitometric analyses (**A**–**D**) are displayed below the respective immunoblot; the respective protein of interest was related to β-actin. Data are presented as mean ± SEM; * *p* < 0.05, **** *p* < 0.0001 (unpaired *t* test); *n* = 5–6/group.

**Figure 3:**
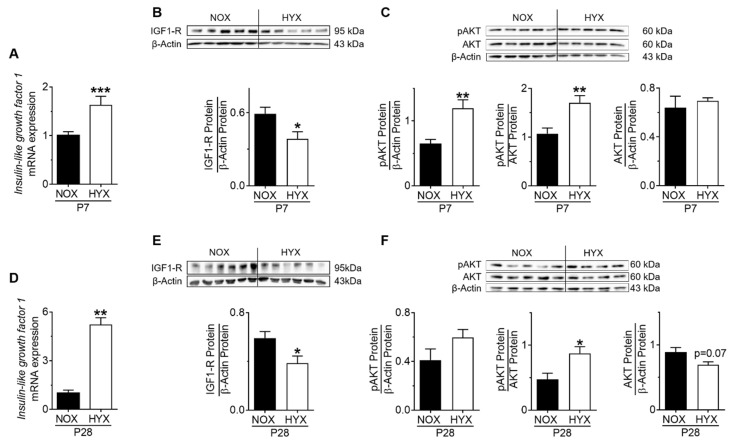
Insulin-like growth factor 1 (IGF1) signaling in lungs of mice exposed to room air (normoxia, NOX, 21% O_2_) or hyperoxia (HYX, 85% O_2_) from birth until postnatal day 7 (P7) or P28. (**A**,**D**) *Igf1* gene expression was assessed in lungs of mice exposed to NOX or HYX at P7 (**A**) and P28 (**D**). (**B**,**E**) Assessment of IGF-1 receptor (IGF1-R) protein abundance in lungs of mice exposed to NOX or HYX at P7 (**B**) and P28 (**E**). (**C**,**F**) Analysis of phosphorylation of AKT protein in lungs of mice exposed to NOX or HYX at P7 (**C**) and P28 (**F**). Densitometric analyses (**B**,**C**,**E**,**F**) are displayed below the respective immunoblot; the protein of interest was related to β-actin; the phosphorylated protein was related to total protein or to β-actin. Data are presented as mean ± SEM; * *p* < 0.05, ** *p* < 0.01, *** *p* < 0.001 (unpaired *t* test); *n* = 5–12/group.

To investigate whether intrinsic-pulmonary GH–IGF1 signaling is dynamically regulated during short-term (P7) and prolonged (P28) hyperoxia (HYX) compared to controls (NOX), we assessed mRNA expression and protein abundance of GH-R and IGF1 as well as downstream mediators STAT5 and AKT, respectively, using RT-PCR and immunoblot. The gene expression of growth hormone receptor (*Ghr*) was significantly reduced after HYX at P7 (Supplementary Figure S1A), whereas GH-R protein abundance was unaltered (Figure 2A). Phosphorylation of STAT5, a downstream modulator of GH, was also decreased at P7 (Figure 2B). While prolonged HYX did not alter the expression of *Ghr* mRNA and GH-R protein in the lung at P28, STAT5 signaling remained significantly reduced by 50% when compared to the control group (Supplementary Figure S1B; Figure 2C,D). Since IGF1 is strongly regulated by GH signaling, we next investigated IGF1/IGF1-R/AKT signaling in the lung. Interestingly, we found an almost two-fold induction of *Igf1* mRNA and a significant increase in phosphorylated AKT, whereas *Igfr1* mRNA and IGF1-R protein were significantly decreased after HYX at P7, most likely as a compensatory mechanism (Figure 3A–C; Supplementary Figure S1D). These findings were also evident in lungs at P28, showing a six-fold induction of *Igf1* mRNA, reduced IGF1-R protein, and activation of AKT signaling (Supplementary Figure S1E, Figure 3D–F). To conclude, short-term and prolonged postnatal HYX inhibits STAT5-signaling, whereas IGF1/AKT signaling is significantly increased in murine lungs.

### 3.3. HYX Regulates the GH–IGF1 Axis during the Regenerative Phase of Lung Injury

**Figure 4:**
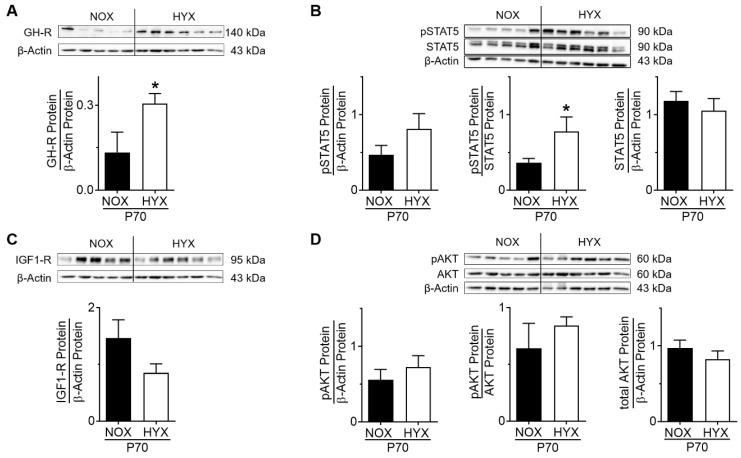
Growth hormone/insulin-like growth factor 1 (GH–IGF1) axis in lungs of mice exposed to room air (normoxia, NOX, 21% O_2_) or hyperoxia (HYX, 85% O_2_) from birth until postnatal day 28 (P28), followed by recovery in room air. (**A**,**C**) GH receptor (GH-R) (**A**) and IGF1 receptor (IGF1-R) (**C**) protein abundance was assessed in lungs at P70. (**B**,**D**) Analysis of phosphorylation of signal transducer and activator of transcription 5 (STAT5) (**B**) and AKT (**D**) protein abundance in lungs of mice exposed to NOX or HYX at P70. Densitometric analyses are displayed below the respective immunoblot; the respective protein of interest was related to β-actin; the phosphorylated protein was related to total protein or to β-actin. Data are presented as mean ± SEM; * *p* < 0.05 (unpaired *t* test); *n* = 5–6/group.

In order to investigate the regulation of the intrinsic-pulmonary GH–IGF1 axis after the regenerative phase, mice were exposed to NOX or HYX for 28 days and recovered in NOX until P70. Subsequently, we assessed gene and protein expression of GH-R and IGF1-R as well as the activation of STAT5 and AKT signaling. At P70, *Ghr* mRNA was not significantly changed (Supplementary Figure S1C), but GH-R protein was markedly increased in lungs of mice exposed to prolonged postnatal HYX when compared to the control (Figure 4A). Along with this finding, phosphorylation of STAT5 was significantly greater in HYX than NOX mice (Figure 4B). At P70, *Igf1r* mRNA expression was significantly decreased and IGF1-R protein slightly decreased in lungs of mice exposed to prolonged postnatal HYX (Supplementary Figure S1F; Figure 4C); phosphorylation of AKT was unaltered in HYX when compared to NOX (Figure 4D). In summary, the regenerative phase after HYX-exposure is associated with an activation of GH-R/STAT5 signaling in murine lungs.

### 3.4. Dysregulation of Lung-Intrinsic GH–IGF1 Signaling Is Associated with Proliferation of αSMA^+^ Cells, Indicative of Myofibroblasts

**Figure 5:**
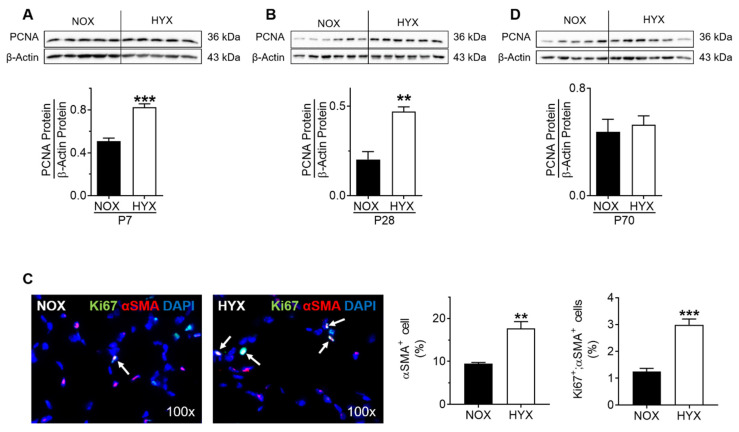
Determining proliferation in lungs of mice exposed to room air (normoxia, NOX, 21% O_2_) or hyperoxia (HYX, 85% O_2_) from birth until postnatal day 7 (P7) or P28, followed by recovery in room air until P70. (**A**,**B**,**D**) Assessment of proliferating cell nuclear antigen (PCNA) protein abundance in lungs at P7 (**A**), P28 (**B**), and P70 (**D**). Densitometric analyses are displayed below the respective immunoblot; the respective protein of interest was related to β-actin. (**C**) Representative co-immunofluorescent staining for αSMA (marker of myofibroblasts) and Ki67 (marker of proliferation) in lungs at P14 as an intermediate stage between P7 and P28. Quantification of relative number of αSMA^+^ and double positive (αSMA; Ki67) cells in %. Data are presented as mean ± SEM; ** *p* < 0.01, *** *p* < 0.001 (unpaired *t* test); *n* = 4–6/group.

Both GH and IGF1 are central in the regulation of proliferative pathways. To assess proliferation, we performed immunoblots for PCNA in total lung homogenate at P7, P28, and P70. Along with activation of IGF1/AKT signaling during short-term (P7) and prolonged (P28) hyperoxia, we found a significant increase of PCNA protein at both time points, indicative of proliferation and possibly related to fibroproliferative processes (Figure 5A,B). We next assessed proliferating myofibroblasts in the lungs at P14, an intermediate stage between P7 and P28, with co-immunofluorescent staining for Ki67 and αSMA as a marker for proliferation and myofibroblasts, respectively. Quantification of the percentage of Ki67 and αSMA positive cells did not only show an almost three-fold increase in proliferating αSMA^+^ cells, but a two-fold increase in the relative number of αSMA^+^ cells as well (Figure 5C). Interestingly, after recovery in room air, unaffected IGF1 signaling was linked to an unaltered PCNA protein abundance in lungs of the HYX group when compared to the control group (Figure 5D).

### 3.5. Alveolar Epithelial Cell Markers Are Dysregulated after HYX in a Time-Dependent Manner

**Figure 6:**
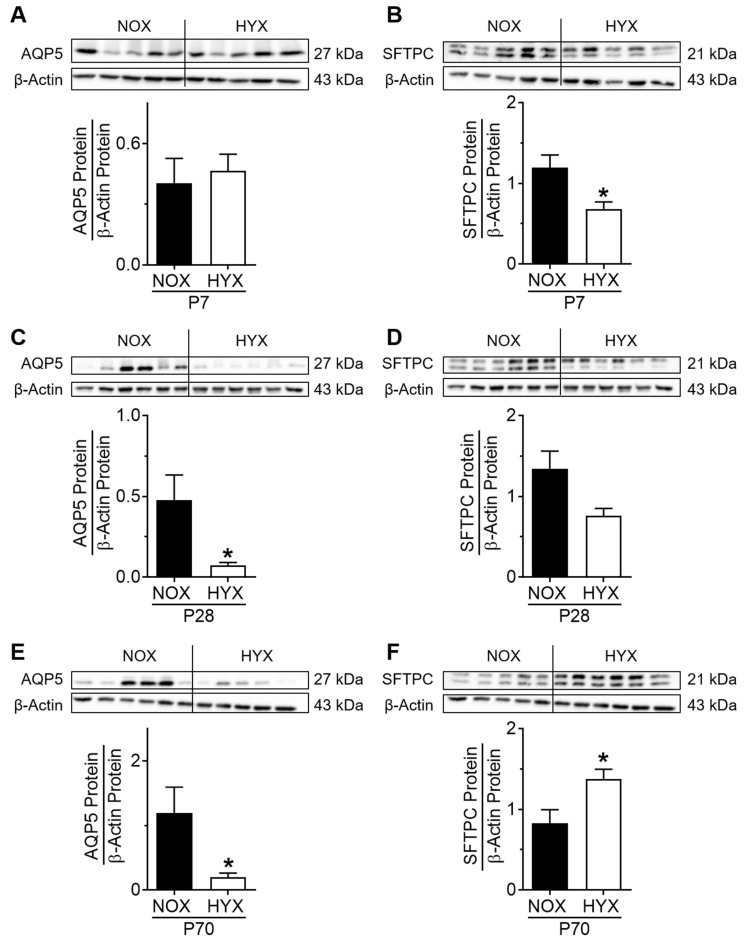
Assessment of alveolar epithelial cell markers in lungs of mice exposed to room air (normoxia, NOX, 21% O_2_) or hyperoxia (HYX, 85% O_2_) from birth until postnatal day 7 (P7) or P28, followed by recovery in room air until P70. (**A**,**C**,**E**) Assessment of Aquaporin 5 (AQP5) protein abundance in lungs of mice exposed to NOX or HYX at P7 (**A**), P28 (**C**), and P70 (**E**). (**B**,**D**,**F**) Assessment of surfactant protein C (SFTPC) protein abundance in lungs of mice exposed to NOX or HYX at P7 (**B**), P28 (**D**), and P70 (**F**). Densitometric analyses are displayed below the respective immunoblot; the respective protein of interest was related to β-actin. Data are presented as mean ± SEM; * *p* < 0.05 (unpaired *t* test); *n* = 5–6/group.

Since impaired alveolar growth is a key characteristic of lungs with BPD, and epithelial cells are central in the process of alveolar growth, we next investigated alveolar epithelial cell markers, including aquaporin 5 (AQP5) as a marker for ATI, and surfactant protein C (SFTPC) as a marker for ATII cells in total lung homogenates of mice exposed to short-term HYX (P7), prolonged HYX (P28), and after recovery in room air (P70). AQP5 protein was not changed at the acute stage, while it was significantly less at P28 and P70, comparing HYX to NOX controls (Figure 6A,C,E). SFTPC protein abundance was significantly decreased at the acute stage and slightly lower at the chronic stage in HYX compared to the control (Figure 6B,D). At P70, we detected an upregulation of SFTPC protein in HYX compared to NOX (Figure 6F). In summary, expression of the ATI marker AQP5 is decreased, and expression of the ATII marker SFTPC is upregulated over time due to hyperoxia exposure.

### 3.6. HYX Regulates GH–IGF1 Signaling in MLE-12

**Figure 7:**
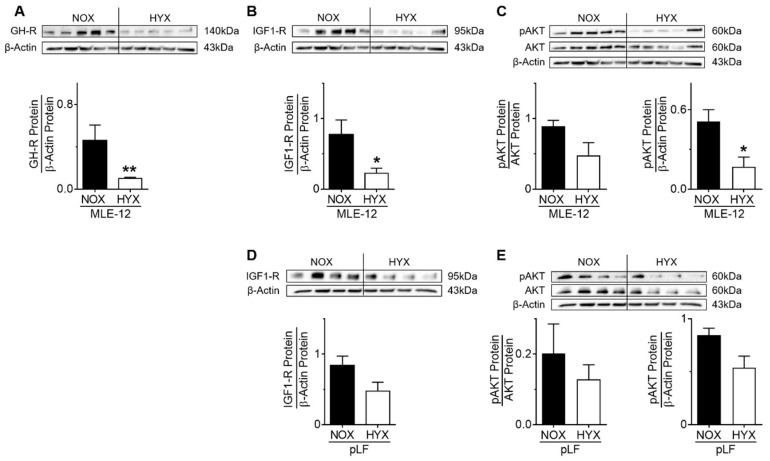
Growth hormone/insulin-like growth factor 1 (GH–IGF1) axis in murine alveolar epithelial cells (MLE-12) and primary lung fibroblasts (pLFs) after exposure to hyperoxia (HYX, 85% O_2_) or normoxia (NOX, 21% O_2_). Protein abundance of GH receptor (GH-R) (**A**), IGF1 receptor (IGF1-R) (**B**), and phosphorylation of AKT (**C**) was assessed after incubation of MLE-12 under NOX or HYX for 48 h. Protein abundance of IGF1-R (**D**) and phosphorylation of AKT (**E**) were measured after exposure of pLFs to NOX or HYX for 48 h. Densitometric analyses are displayed below the respective immunoblot; the respective protein of interest was related to β-actin. Data are presented as mean ± SEM; * *p* < 0.05, ** *p* < 0.01 (unpaired *t* test); *n* = 4–5/group.

Since impaired GH–IGF1 signaling and a decrease of ATII markers was observed in murine lungs during the acute phase of HYX, we next investigated GH–IGF1 signaling in MLE-12 exposed to NOX (21% O_2_) or HYX (85% O_2_) in vitro. Exposure of murine MLE-12 to HYX diminished protein abundance of GH-R, IGF1-R, and phosphorylated AKT compared to NOX (Figure 7A–C). Similarly, we exposed pLFs to NOX and HYX and found a mild but not significant reduction of IGF1-R protein and pAKT signaling (Figure 7D,E). We can therefore conclude that HYX cell-specifically inhibits GH–IGF1 signaling in MLE-12, but not in pLFs.

### 3.7. GH-R and IGF1-R Regulate STAT5 and AKT Signaling as Well as Gene Expression and Homeostasis of MLE-12 and pLFs

**Figure 8:**
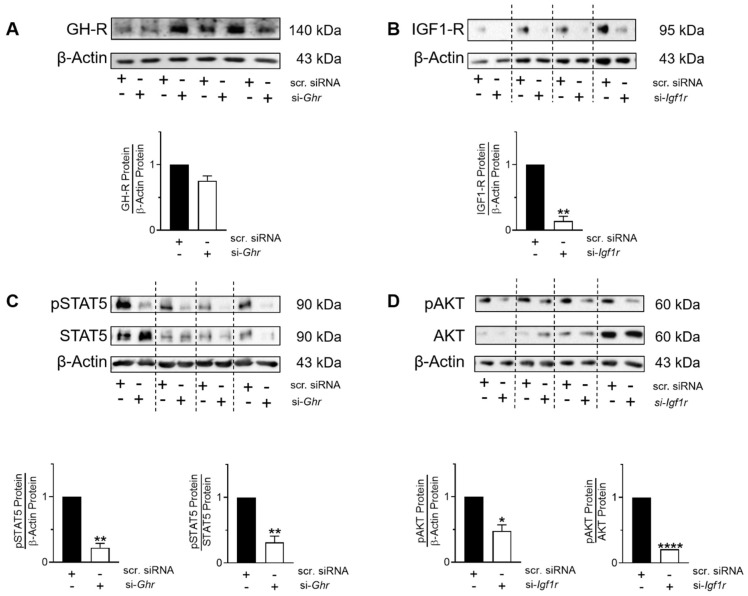
(**A**,**B**) Silencing of growth hormone receptor (GH-R) (**A**) and insulin growth factor 1 receptor (IGF1-R) (**B**) in cultured rat primary neonatal fibroblasts (pnF) using the siRNA technique. Rat pnF cells were transfected with either anti Ghr-siRNA (si-*Ghr*), anti Igf1r-siRNA (si-*Igf1r*), or scrambled siRNA (scr. siRNA) for 4 h. Cells were maintained in serum-rich medium and harvested after 48 h or 72 h for protein isolation in three to four independent experiments. Immunoblots show phosphorylated STAT5 (pSTAT5) and total STAT5 (**C**) as well as phosphorylated AKT (pAKT), and total AKT (**D**) after silencing of *Ghr* or *Igf1r*, respectively. Densitometric analyses are shown under the respective immunoblot. The phosphorylated protein was related to total protein or the loading control to β-actin. Total proteins are related to β-actin. Data are presented as mean ± SEM; * *p* < 0.05, ** *p* < 0.01, **** *p* < 0.0001 (paired *t* test); *n* = 3–4/group.

In order to test if GH-R and IGF1-R indeed regulate STAT5 and AKT signaling, respectively, we silenced *Ghr* and *Igf1r* in pnF using the siRNA technique. After 48 h and 72 h of transfection, we assessed protein expression and phosphorylation of STAT5 and AKT in pnF with immunoblot. The analysis revealed that silencing GH-R and IGF1-R blocks the phosphorylation of STAT5 and AKT in pnF, respectively (Figure 8C,D).

**Figure 9:**
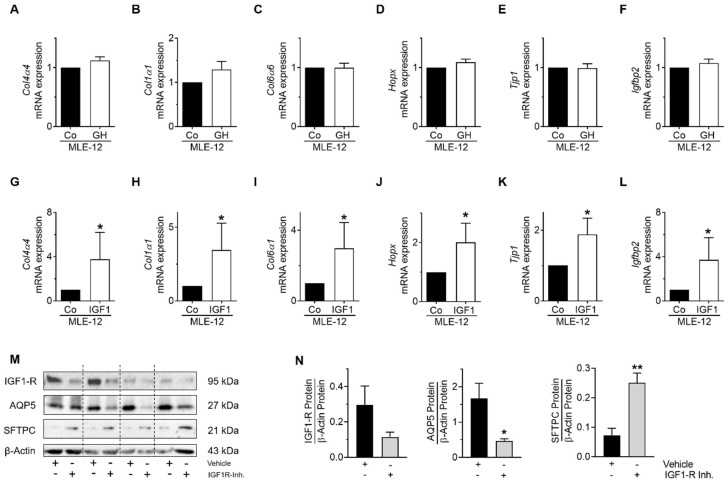
Analysis of epithelial and mesenchymal cell markers in MLE-12 after stimulation with growth hormone (GH) or insulin growth factor 1 (IGF1). MLE-12 were stimulated for 24 h with either GH (100 nmol/L) or IGF1 (10 ng/mL) and their respective vehicles (10 mmol sodium bicarbonate or dH_2_O), and gene expression of collagen 4α4 (*Col4a4*) (**A**,**G**), collagen 1α1 (*Col1a1*) (**B**,**H**), collagen 6α1 (*Col6a1*) (**C**,**I**), homeodomain-only protein (*Hopx*) (**D**,**J**), zonula occludens 1 (tight junction protein-1, *Tjp1*) (**E**,**K**), and IGF binding protein 2 (*Igfbp2*) (**F**,**L**) was determined. (**M**,**N**) IGF1-R was blocked using an inhibitor (20 nM/mL, respective vehicle ethanol), and protein abundance of surfactant protein C (SFTPC), aquaporin 5 (AQP5), and IGF1-R were assessed with immunoblot. Densitometric analyses are shown next to the immunoblots. Total protein was related to the loading control β-actin. Data are presented as mean ± SEM; * *p* < 0.05, ** *p* < 0.01 (unpaired *t* test); *n* = 4–6/group.

Next, to study the functional impact of GH and IGF1 on MLE-12, gene expression of mesenchymal and epithelial cell markers was assessed after stimulation with either GH or IGF1. Stimulation of MLE-12 with GH had no effect on mesenchymal cell marker expression (Figure 9A–F), whereas stimulation with IGF1 resulted in an increase of mesenchymal cell markers including *Col4a4*, *Col1a1*, and *Col6a1* as well as alveolar epithelial cell markers such as *Hopx*, *Tjp1*, and *Igfbp2* compared to MLE-12 exposed to the respective vehicles (Figure 9G–L). To further strengthen the notion that IGF1-R signaling regulates differentiation of MLE-12, we treated the cells with an IGF1-R inhibitor. Blockade of IGF1-R caused a three-fold increase of SFTPC protein abundance and a reduction of AQP5 by more than 50%, supporting the important role of IGF1-R signaling in maintenance of lung epithelial cell homeostasis (Figure 9M,N). Thus, IGF1 regulates mesenchymal and alveolar epithelial cell markers in MLE-12, while GH has no effect.

**Figure 10:**
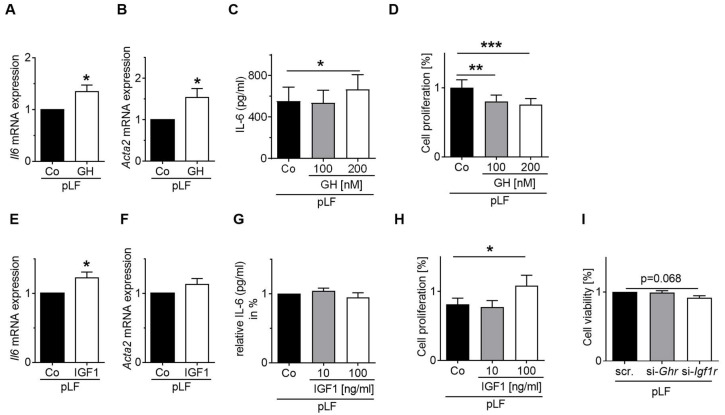
Functional role of growth hormone (GH) and insulin-like growth factor (IGF1) on proliferation and activation of primary neonatal lung fibroblasts (pLFs). (**A**–**F**) pLFs were stimulated for 24 h with either GH (100 nmol/L) or IGF1 (10 ng/mL), and their respective vehicles (10 mmol sodium bicarbonate or dH_2_O) and gene expression of interleukin 6 (*Il6*) (**A**,**E**) and αSMA (*Acta2*) (**B**,**F**) was analyzed. (**C**,**G**) Measurement of IL-6 protein abundance in cell supernatant using ELISA. (**D**,**H**) Assessment of proliferation in pLFs after stimulation for 24 h with GH (100 nmol/L or 200 nmol/L) or IGF1 (10 ng/mL or 100 ng/mL) using BrdU assay. (**I**) Analysis of viability of pLFs after treatment with IGF1-R inhibitor (20 nM/mL, respective vehicle ethanol) using the MTT assay. Data are presented as mean ± SEM. * *p* < 0.05, ** *p* < 0.01, *** *p* < 0.001 (unpaired *t* test or RM one-way ANOVA with Bonferroni post-test); *n* = 4–6/group.

Finally, we studied the functional role of GH and IGF1 in pLFs. On the mRNA level, stimulation with GH increased *Il6* and the myofibroblast marker *Acta2* mRNA (Figure 10A,B). The expression of the fibroblast marker *Vimentin* was reduced, and *Collagen 4α4* mRNA was not affected (Supplementary Figure S2A). On the protein level, IL-6 was increased in the supernatant of GH-treated pLFs, but not in MLE-12 (Figure 10C, Supplementary Figure S3A). Moreover, proliferation was assessed by BrdU assay and revealed that stimulation with GH reduced proliferative capacity of pLFs when compared to pLFs treated with the respective vehicles (Figure 10D). In contrast, stimulation of pLFs with IGF1 increased *Il6* mRNA, but had no effect on *Acta2*, *Vimentin*, or *Collagen 4α4* (Figure 10E,F, Supplementary Figure S2B). Interestingly, IL-6 protein in pLF supernatant was reduced, possibly through altered translation or increased degradation; IL-6 protein in MLE-12 was not changed by IGF1 (Supplementary Figure S3B). Furthermore, stimulation with IGF1 and blockade of IGF1-R increased proliferation and reduced viability, respectively (Figure 10H,I).

## 4. Discussion

The present study investigates the dynamic regulation of the GH–IGF1 axis in murine lungs after short-term (P7) and prolonged postnatal hyperoxia (P28) as well as after recovery in normoxia, at P70. The in vivo findings are in part complemented by in vitro studies using cultured MLE-12 as well as pLFs. Our data demonstrate a marked dysregulation of the IGF1/AKT signaling machinery during short-term and prolonged exposure to HYX, but not during lung recovery in room air. In contrast, GH–STAT5 signaling was oppositely regulated when compared to IGF1-R/AKT in the phases described, indicating a possible lung-intrinsic uncoupling of GH and IGF1 signaling. Furthermore, cell culture studies showed a major impact of IGF1, but not of GH, on the differentiation of MLE-12. Interestingly, only GH induced significant expression of IL-6 protein and reduced proliferation of pLFs, whereas IGF1 had an opposite effect. Thus, our data provide evidence that the GH–IGF1 axis is important in the regulation of differentiation and proliferation of alveolar epithelial cells and neonatal lung fibroblasts. Disruption of these signaling pathways in neonatal lungs after hyperoxia could account for an impaired alveolarization and regeneration, as seen in infants with BPD.

Advances in neonatal management of preterm infants over the last decades, including application of surfactant proteins and the avoidance of mechanical ventilation and oxygen supply, have improved survival rates. These medical innovations along with lower gestational age have shifted the ‘old’ BPD to the ‘new’ BPD. While lungs of infants with the ‘old’ BPD are characterized by fibroproliferative processes, thickened septae and reduced alveolarization, the ‘new’ BPD mainly results in alveolar simplification and capillary hypoplasia [19]. To investigate the pathomechanisms of BPD, various animal models have been established over the last decades, including exposure of newborn mice to prolonged hyperoxia [19,20]. Increased oxygen levels impair alveolarization and perturb matrix remodeling favoring fibrosis. Recent studies show that length of exposure and the concentration of oxygen determine the level of injury. While 40% O_2_ for a short period induces an arrest of alveolarization, matrix remodeling is only mildly affected. In contrast, to study both reduced alveolar growth and marked pro-fibrotic processes, higher levels of O_2_ are required for a prolonged period (20). Since we aimed to investigate the GH–IGF1 axis in impaired alveolarization and matrix remodeling of the newborn as a model for the changes seen in infants with severe BPD [18], we used 85% O_2_ for a prolonged period in neonatal mice, inducing characteristic features of BPD [14,15].

GH–IGF1 signaling is a central signaling pathway that stimulates growth, cell reproduction, and cell regeneration. GH is a peptide hormone that is synthesized, stored, and secreted by somatotropic cells within the lateral wings of the anterior pituitary gland and promotes long bone growth, the regulation of carbohydrate and lipid metabolism, liver metabolic function, as well as energy balance. GH signals through a cell surface receptor, known as growth hormone receptor (GH-R), resulting in the activation of several intracellular signaling pathways, e.g., STAT5, and expression of a diverse set of genes, which enables GH to exert its pleiotropic effect on cell growth, differentiation, and apoptosis [21,22,23,24,25,26,27]. The effects of GH are mediated in part through IGF1, which is produced in the liver and other tissues in response to GH. IGF1 binds to its receptor IGF1-R, activating a receptor tyrosine kinase and initiating AKT signaling [22,23]. Interestingly, there is a growing body of evidence that GH and IGF1 are not only produced by the pituitary gland and the liver, respectively, but also in the lung, pointing towards a lung-intrinsic GH–IGF1 axis that could promote anabolism as well [5]. This notion was supported by prior studies from our group showing gene expression of GH and IGF1 in the developing rat lung, suggesting an important role for both factors in lung development. Several studies have revealed that IGF1 signaling deficiency results in disrupted lung architecture in animal models and alveolar hyperplasia in humans [6,8,28,29]. This is further supported by studies using transgenic mice with deletion of *Igf1*. Mutant mice with a deficiency of *Igf1* are less viable due to a severe alveolar hypoplasia and neonatal respiratory distress [30]. Similarly, initial studies have indicated a role for GH and/or local autocrine/paracrine actions of GH produced within the lung in early lung growth, oxidative protection, lipid and energy metabolism, as well as in proteasomal activity. Both the overexpression of GH as well as the knockout of GH-R impact the proteomic profile of the lung and pulmonary matrix composition, suggesting a crucial role during alveolar development [24,25,31]. Our present study confirms a lung-intrinsic GH–IGF1 signaling axis, not only during normal development, but also in aberrant lung development.

The results of the present study show a discrepancy between mRNA and protein expression for both the ligand and receptor. For example, we found increased *Igf1* mRNA, whereas IGF1-R protein was reduced at P7 and P28. The mRNA expression is not predictive for protein abundance. This can be caused by various cellular and molecular mechanisms, including post-transcriptional regulation, alterations of the protein half-life, and modifications of degradation processes [32]. In addition, miRNAs and the oxidative stress response are other mechanisms by which protein degradation might be affected and could contribute to differences between mRNA expression and protein abundance [33,34]. Recent studies suggest an escape mechanism of cells under oxidative stress by reducing the activity of ATP-dependent processes. To protect cells against the accumulation of irreversible damaged protein aggregates, a number of ROS- mediated changes occur that can contribute to reduction of new protein synthesis [35]. These studies can explain in part why the mRNA expression in our study is not consistent with protein abundance.

Several studies demonstrate a mechanistic role for IGF1 in acute and chronic lung diseases, including acute respiratory distress syndrome (ARDS) with an upregulation of IGF1 and IGF1-R expression, but also inflammatory as well as fibrotic conditions [29,36]. For example, free IGF1 is significantly elevated in early ARDS [37]. Moreover, IGF1 expression and/or signaling is upregulated in patients with idiopathic fibrotic lung diseases and could regulate myofibroblast activation [10,11,12] and the epithelial to mesenchymal transition [38]. Here, we show that GH induces the expression of *Il6* and IL-6 protein and regulates proliferation of primary neonatal fibroblasts, confirming the important role of these factors in triggering fibrotic processes.

Cell type-specific and developmentally regulated expression of growth factors and their concerted interaction during a restricted time period is very important. Prior studies from our group along with other reports demonstrate an important functional role of GH–IGF-1 signaling in perinatal lung injury, including intrauterine growth restriction (IUGR) and associated prematurity [4]. While risk factors are well described, the mechanisms, including disruption of critical signaling pathways, remain incompletely understood. Interestingly, preterm infants show a marked reduction of IGF1 levels, similar to an IGF1 deficiency [39]; IGF1 has been implicated in the etiology of BPD. The present study investigated the impact of hyperoxia on neonatal lung-intrinsic IGF1 signaling and in particular on the GH–IGF1 axis. While GH signaling was reduced in the lung during short-term and prolonged exposure to hyperoxia, IGF1 signaling was markedly activated. Interestingly, after recovery in normoxia, we found an activation of GH-signaling, indicating a possible role in regeneration [40,41,42,43,44,45]. These findings suggest a possible uncoupling of the classical GH–IGF1 axis and differentially regulated functions of GH and IGF1 in neonatal hyperoxia-induced lung injury. Recent experimental studies show that recombinant human (rh) IGF1 (rhIGF1)/BP3 enhances lung growth in experimental BPD, supporting the notion that IGF1 could be central in promoting regeneration [13]. Ongoing clinical trials test rhIGF1/rhIGFBP-3 for prevention of BPD and show that the occurrence of severe BPD is decreased [46].

Lung growth arrest and reduced alveolar regeneration are key characteristics of lungs with BPD. ATII cells have the capacity to self-renew and to differentiate into ATI cells. After acute injury, ATIIs give rise to ATIs, promoting regeneration of alveoli. In our study, we show a significant loss of ATII (SFTPC) and ATI (AQP5) markers, indicating a loss of alveolar epithelial cells after short-term and prolonged hyperoxia. Interestingly, while the abundance of SFTPC is significantly increased after recovery, AQP5 remains reduced by more than 50%, indicating a possible disrupted ATII to ATI differentiation along with altered GH–IGF1-signaling. IGF1-related effects are linked to proliferation and differentiation of various pulmonary cell types such as club cells, airway basal cells, alveolar epithelial cells, and fibroblasts [6]. For example, there is evidence that IGF-1 signaling regulates basal cell differentiation and induces expression of basal cell markers [47,48]. These findings indicate that IGF1 expression in lung epithelial cells is crucial for controlling basal cell fate and organ development. Moreover, studies have pointed out that the differentiation of basal cells is mediated via an activation of IGF1-R-AKT signaling [49]. In addition, blockade of IGF1 or IGF1-R inhibits ATII proliferation and trans-differentiation, whereas recombinant IGF1 promotes differentiation of ATIIs to ATIs [50]. Here, we show that treatment of MLE-12 with IGF1 disrupts epithelial cell differentiation and could thereby promote trans-differentiation of ATIIs in vitro, promoting lung growth. Collectively, our data coupled with recent studies indicate that GH–IGF1 signaling is a promising target to treat BPD.

**Figure 11:**
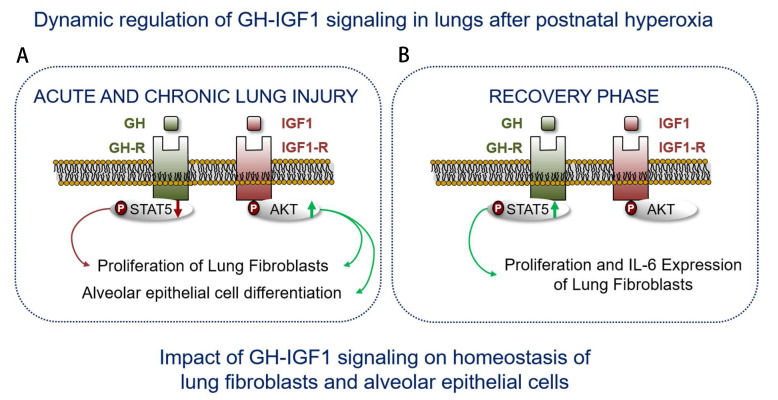
The scheme illustrates regulation of the growth hormone (GH) and insulin-like growth factor 1 (IGF1) system in murine lungs after short-term (postnatal day 7, P7) and prolonged (P28) exposure to hyperoxia (HYX, 85% O_2_) from P1 to P28, leading to acute and chronic lung injury (**A**), and after the recovery phase in room air from P28 to P70 (**B**). First, we show an inhibition and activation of GH-R/STAT5 and IGF1/AKT signaling, respectively, in neonatal lungs during short-term and prolonged HYX. These findings suggest a possible uncoupling of the classical GH–IGF1 axis. Second, GH-STAT5 signaling is activated after the recovery phase, whereas IGF1-AKT is unaltered. These findings in vivo along with our cell culture studies indicate a functional role of GH–IGF1 signaling cascades in neonatal lung injury and regeneration with an emphasis on alveolar epithelial cell and fibroblast biology.

In summary, prematurity is related to reduce activation of the GH–IGF1 axis and poor weight gain, retinopathy, as well as BPD. The present study proposes a novel possible uncoupling of the GH–IGF1 axis in lungs of neonatal mice exposed to short-term and prolonged hyperoxia (Figure 11). We used a global in vivo approach investigating whole lung homogenate and a specific in vitro approach using MLE-12 and pLFs. Treatment with IGF1 after injury could promote regeneration and alveolar growth. The pharmacological amenability of IGF1 offers new therapeutic avenues for lung recovery.

## Figures and Tables

**Table 1:** Mean value ± standard error of the mean (SEM) of body and lung weight (g).

	P7		P28		P70	
	NOX	HYX	NOX	HYX	NOX	HYX
Body weight (g)	3.576 ± 0.113	3.455 ± 0.137	13. 90 ± 0.328	9.469 ± 0.154 ****	25.220 ± 0.944	23.780 ± 1.494
Lung weight (g)	0.076 ± 0.004	0.062 ± 0.003 *	0.072 ± 0.002	0.077 ± 0.002 *	0.081 ± 0.003	0.199 ± 0.103

Values are displayed as mean ± SEM; * *p* < 0.05; **** *p* < 0.0001.

## Data Availability

Not applicable.

## Supplementary Materials

The following are available online at https://www.mdpi.com/article/10.3390/cells10112947/s1, Figure S1: Gene expression of growth hormone receptor (*Ghr*) and insulin-like growth factor 1 receptor (*Igf1r*) in murine lungs of mice exposed to room air or hyperoxia from birth until postnatal day 7 (P7) or P28. Figure S2: Functional role of Growth Hormone (GH) and Insulin-like growth factor (IGF1) in gene expression of primary neonatal lung fibroblasts (pLF). Figure S3: Functional role of Growth Hormone (GH) and Insulin-like growth factor (IGF1) on IL-6 secretion of murine lung epithelial cells (MLE-12). Table S1: List of primers used for real-time RT-PCR.
