# Supplementary material for: Dynamic Regulation of GH–IGF1 Signaling in Injury and Recovery in Hyperoxia-Induced Neonatal Lung Injury
Source: Cells. 2021 Oct 29;10(11):2947. doi: 10.3390/cells10112947 (PMC8616454; doi:10.3390/cells10112947)
Supplement: Supplementary file 1 [file cells-10-02947-s001.zip › cells-1405682-supplementary.pdf]

Supplementary Materials:

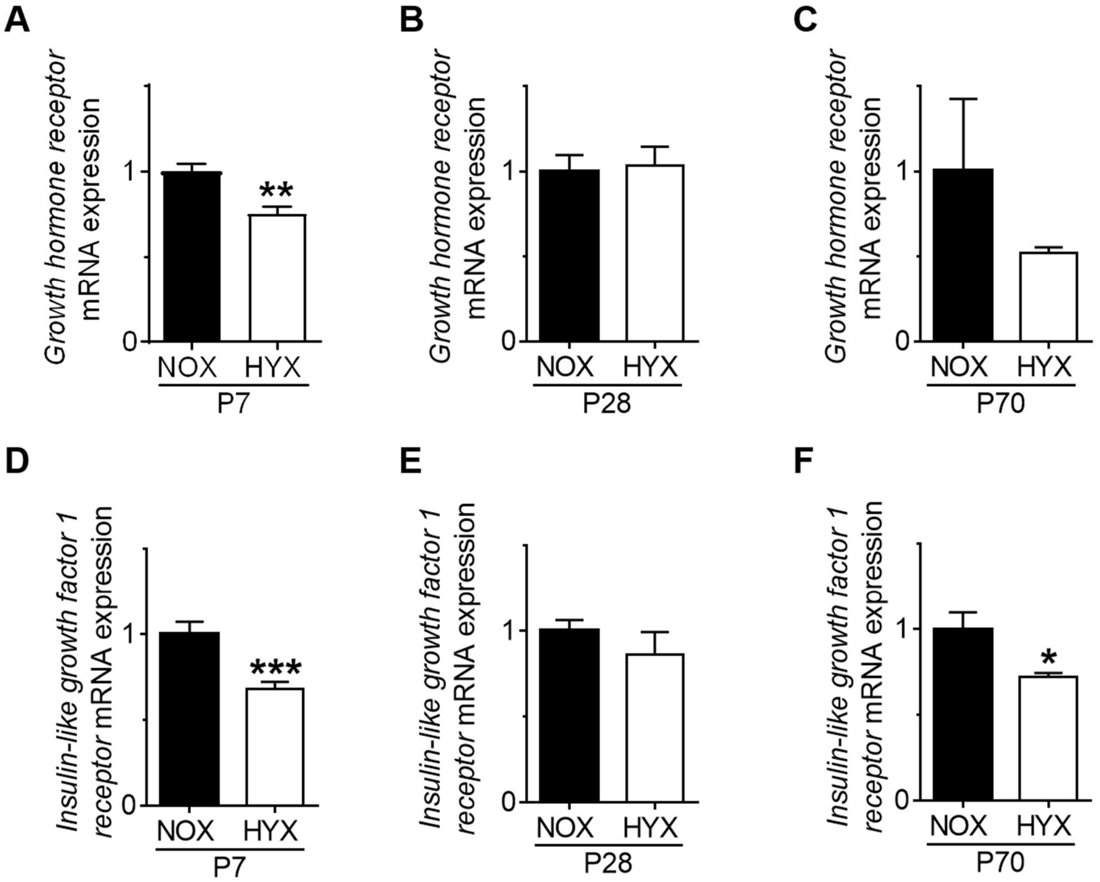

Supplementary Figure S1. Gene expression of growth hormone receptor (*Ghr*) and insulin-like growth factor 1 receptor (*Igf1r*) in murine lungs of mice exposed to room air (normoxia, NOX, 21 % O_2) or hyperoxia (HYX, 85 % O_2) from birth until postnatal day 7 (P7) or P28 using qRT-PCR. A-C: Measurement of lung *Ghr* mRNA at P7 (A), P28 (B), and P70 (C). D-F: Assessment of lung *Igf1r* mRNA at P7 (D), P28 (E), and P70 (F); β-Actin served as housekeeping gene. Data are presented as mean±SEM. *p<0.05, **p<0.01, ***p<0.001 (Unpaired t test); n=10-12/group.

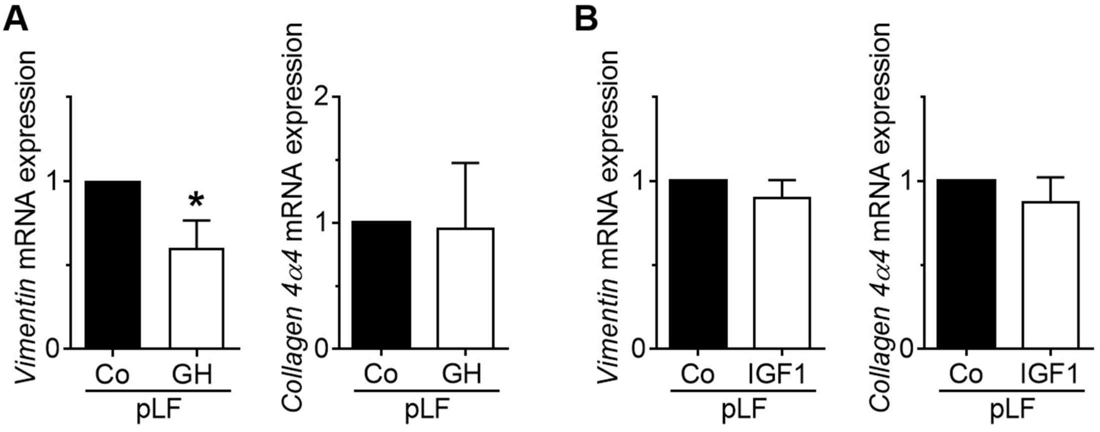

Supplementary Figure S2. Functional role of Growth Hormone (GH) and Insulin-like growth factor (IGF-1) in gene expression of primary neonatal lung fibroblasts (pLF). A, B: pLF were stimulated for 24 h with either GH (100 nmol/l) (A) or IGF-1 (10 ng/ml) (B), and their respective vehicles (10 mmol sodium bicarbonate or dH_2O). Gene expression of Vimentin (*Vim*) and Collagen 4α4 (*Col4a4*) was analyzed; β-Actin served as housekeeping gene. Data are presented as mean±SEM. *p<0.05 (Unpaired t test); n=6/group.

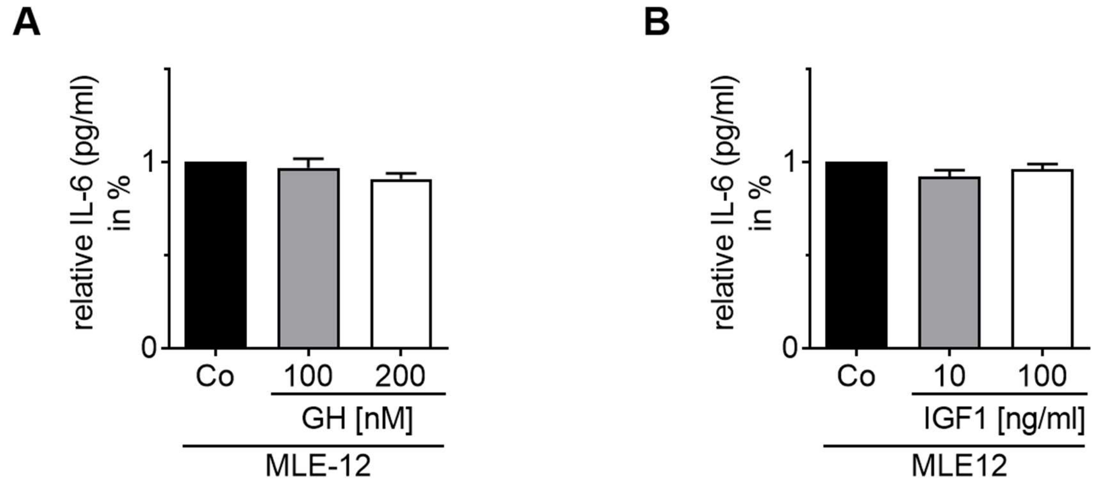

Supplementary Figure S3. A, B. Functional role of Growth Hormone (GH) (A) and Insulin-like growth factor (IGF-1) (B) on IL-6 secretion of murine lung epithelial cells (MLE-12). IL-6 protein abundance in cell supernatant was measured with ELISA, data are presented as mean±SEM. (RM One-way ANOVA with Bonferroni post-test); n=4/group.

Supplementary Table S1. List of primers used for real-time RT-PCR; *SYBR-Green primer.

A-smooth muscle actin (Acta 2)	Forward, ACATCAGGGAGTAATGGTTGGAAT
	Reverse, GGTGCCAGATCTTTTCCATGTC
	Probe (FAM), CGATAGAACACGGCATCATCACCAACTG (TAMRA)
**Actb*	Forward, TGACAGGATGCAGAAGGAGATTACT
	Reverse, GCCACCGATCCACACAGAGT
Actb	Forward, TGACAGGATGCAGAAGGAGATTACT
	Reverse, GCCACCGATCCACACAGAGT
	Probe (FAM), ATCAAGATCATTGCTCCTCCTGAGCGC (TAMRA)
**Collagen 1a1 (Col1a1)*	Forward, GCAGTGCTGTTGCGATCTTG
	Reverse, CAGAGGGACAGAGCACAGCTT
Collagen 4a4 (Col4a4)	Forward, GAAGGCGCACAATCAAGATCT
	Reverse, CACACTTGGTGGATGTTGCAGTA
	Probe (FAM), CTTCCTGTGTTTAGCACTCTGCCCTTTGC
**Collagen 6a1 (Col6a1)*	Forward, GGTGGCCACACAGGACATC
	Reverse, GGTGTCGAGCACGAAGAATAGA
**Growth hormone receptor (Ghr)*	Forward, CAAAAATGTTTCACTGTTGACGAAA
	Reverse, GAATCCCGGTCAAACTAATGTTTAG
	Forward, CGGAGGACCAGGTGGAGAT

**Homeodomain-only protein homeobox (Hopx)*	Reverse, GCGCTGCTTAAACCATTTCTG
**Insulin growth factor binding protein 2 (Igfbp2)*	Forward, GCCCCCTGGAACATCTCTACT
	Reverse, AGAGACATCTTGCACTGCTTAAGGT
Insulin-like growth factor 1 (Igf1)	Forward, GCTGGTGGATGCTCTTCAGTT
	Reverse, GGTGCCCTCCGAATGCT
	Probe (FAM), TTTTACTTCAACAAGCCCACAGGCTATGGC
**Insulin-like growth factor 1 receptor (Igf1r)*	Forward, GCTTCCTGTGAAAGTGATGTTCTC
	Reverse, CGTTTTTAAATGGTGCCTCCTT
Interleukin 6 (Il6)	Forward, ACAAGTCGGAGGCTTAATTACACAT
	Reverse, AATCAGAATTGCCATTGCACAA
	Probe (FAM), TCTTTTCTCATTTCCACGATTTCCCAGAGAA
**Vimentin (Vim)*	Forward, CCCTGAACCTGAGAGAAACTAACC
	Reverse, GTCTCATTGATCACCTGTCCATCT
**Zonula occludens 1 (Tjp1)*	Forward, CCTGTCCCTCAGAGTCAGTTTAGTG
	Reverse, CAATATCTTCGGGTGGCTTCA
